# Can old drugs learn new tricks? Achieving registration and public subsidy listing for off‐patent medicines with novel therapeutic applications

**DOI:** 10.1111/imj.16159

**Published:** 2023-07-06

**Authors:** Jonathan Brett, Dilara Bahceci, Wendy Lipworth, Paul Liknaitzky, Ric O. Day, Anthony Rodgers

**Affiliations:** ^1^ Department of Clinical Pharmacology and Toxicology St Vincent's Hospital Sydney Sydney New South Wales Australia; ^2^ Medicines Intelligence Centre for Research Excellence University of New South Wales Sydney New South Wales Australia; ^3^ The George Institute for Global Health University of New South Wales Sydney New South Wales Australia; ^4^ Department of Philosophy Macquarie University Sydney New South Wales Australia; ^5^ Department of Psychiatry, School of Clinical Sciences Monash University Melbourne Victoria Australia; ^6^ Turner Institute for Brain and Mental Health, School of Psychological Sciences Monash University Melbourne Victoria Australia

**Keywords:** repurposing, generic, medicine, off‐label, off‐patent

## Abstract

With the increasing costs of drug development, repurposing of low‐cost medicines for new indications has never been more important. However, there are multiple barriers to repurposing, particularly for off‐patent medicines, and limited incentives for the pharmaceutical industry to sponsor registration and public subsidy listing. Here, we explore these barriers and their consequences and provide examples of successful repurposing strategies.

Repurposing involves identifying new therapeutic uses for existing medicines. Here, we review some of the challenges and opportunities related to medicine repurposing in Australia, including those related to regulatory and reimbursement approval, and illustrate these with several case examples.

## The importance of medicine repurposing

The imperative for medicine discovery during COVID‐19 has re‐highlighted the benefits and pitfalls of medicine repurposing.^1^ Since little or no new work on safety or formulation is required, the cost, time and risk of development can be radically reduced, compared to the development of New Chemical Entities or New Biological Entities. This is particularly salient in an era of increasing medicine development costs and diminishing success of candidate molecules.^2^ The last decade has seen Big Pharma and biotech introduce groundbreaking innovations in high‐cost, uncommon (low volume) conditions, resulting in an increasingly unmet need for new approaches to common conditions. Hence, the case for repurposing is particularly strong for high‐volume, low‐unit price medicines relevant to common conditions. There are several consequences associated with barriers to medicine repurposing.

### Off‐label prescribing

Formal repurposing is also preferable to *ad hoc* off‐label prescribing, which often arises after initial regulatory approval of a medicine, as more experience is gained, and evidence is generated for off‐label indications. The quality of off‐label use varies from appropriate guideline‐concordant use, such as rosuvastatin and bisoprolol use following myocardial infarction, to use for indications that may be harmful or wasteful, such as quetiapine for behavioural and psychological symptoms of dementia. Although the Therapeutic Goods Administration (TGA) updates product information in response to safety concerns, updates for indications do not otherwise occur unless a sponsor is requesting a new indication. There are also medico‐legal risks to prescribers and safety risks to patients as off‐label prescribing occurs in the absence of systematic pharmacovigilance.

### Equity of access

There are also serious threats to access and equity when medicines are not registered as this is a necessary step prior to Pharmaceutical Benefits Scheme (PBS) listing for public subsidy. For higher‐cost generic medicines, such as rituximab, substantial costs associated with off‐label prescribing are borne by either hospitals or patients, with substantial variability in ability or willingness to pay. Furthermore, off‐label indications for existing registered medicines may be listed in state‐wide formularies, creating a two‐tiered system and inequities between states.^3^ Hence, we believe that both TGA registration and PBS listing are important steps to successfully repurposing a medicine since, without the latter, substantial inequities in access could be introduced for higher‐cost medicines.

### Financial implications

A recent example that demonstrates the economic implications of failing to repurpose a generic medicine are the events surrounding the regulation of esketamine for treatment‐resistant depression. Ketamine has a long history of use as an off‐patent anaesthetic medicine and exists as a racemate of R‐ and S‐ketamine. Initial investigator‐led trials of racemic ketamine for depression demonstrated potential for this indication. However, public funding for this research mostly resulted in small trials, with little to no co‐ordination across funding bodies and no strategic focus on the trials needed for obtaining regulatory approval or reimbursement. Meanwhile, a pharmaceutical company progressed intranasal S‐ketamine (esketamine) through registration by the TGA and over 40 other jurisdictions and are in the process of applying for PBS listing. A recent meta‐analysis suggests that generic ketamine is at least as effective and tolerable for use in depression as esketamine^4^; however, the cost of esketamine is over 30 times higher. Failing to fund trials towards regulatory approval for generic racemic ketamine for depression has resulted in substantial excess costs to patients and possibly governments, with apparently no greater clinical benefit than generic ketamine. However, a sponsor would still be required for generic ketamine, meaning they would need to be appropriately incentivised.

Moreover, even when a medicine is registered on the Australian Register of Therapeutic Goods, it may fail to attract public subsidy simply because a submission for funding cannot be or has not been made. For instance, disulfiram is a TGA‐registered medicine for alcohol dependence, but is not PBS subsidised.^5^ Several attempts have been made by academics and clinicians to collate the evidence required for public subsidy, given the clinical need. However, the resources required to meet the extensive application requirements and demonstrate cost‐effectiveness have not been available, and so the process has faltered. Lack of subsidy means that the medicine costs are borne directly by patients, who are frequently already in financial distress.^5^ This has also led to regular supply disruptions for disulfiram, forcing patients to access unregistered formulations through the TGA special access scheme. This underscores the importance of PBS listing as part of repurposing to ensure equitable and continuous access.

### Old compounds never approved

Although repurposing predominantly refers to obtaining regulatory approval of new indications for old medicines, it is also potentially important for long‐used compounds that have never been registered. Examples include psilocybin‐assisted psychotherapy for depression, and 3,4‐Methylenedioxymethamphetamine (MDMA)‐assisted psychotherapy for post traumatic stress disorder (PTSD). The recent ‘psychedelic renaissance’ has created incentives for the pharmaceutical industry to reformulate or modify the delivery of older compounds such as MDMA and psilocybin to obtain patents. This is particularly salient given the recent TGA rescheduling of MDMA and psilocybin from schedule 9 poisons to schedule 8 controlled substances to treat PTSD and treatment‐resistant depression. Efforts to patent these compounds have been met with multiple legal challenges on the basis that many such patents add negligible clinical value, attempt to monopolise well‐known old medicines and undermine affordable and equitable access.^6^

## Overcoming the barriers to medicine repurposing

To ensure TGA registration for a new indication (a necessary step prior to subsidy), a sponsor must apply for an extension of indication, ensure post‐market surveillance and guarantee supply. This comes at substantial cost, so most applications occur where the medicine is on‐patent to allow for patent extensions. For example, rituximab was initially approved for non‐Hodgkin lymphoma but subsequently repurposed for a range of other cancers and autoimmune conditions originating from B‐cell dysfunction.^7^ However, after a patent ceases, there are few financial incentives for industry to sponsor the addition of new indications to those of established medicines.

### Appropriately incentivising industry

In recognition of the barriers to repurposing, the Department of Health (DoH) recently undertook consultations to identify potential solutions.^8^ As a result, patient, clinician, academic and industry stakeholders identified the need for financial incentives to address the substantial costs associated with medicine registration; for instance, government funding or technical support to assist with submissions to the TGA and subsequently Pharmaceutical Benefits Advisory Committee (PBAC), simultaneous regulatory and reimbursement evaluations, fee reductions and potential exclusivity periods for sponsors.^8^ In addition, stakeholders suggested that the DoH should work in partnership with sponsors by helping to collate published and unpublished data required for submissions, as well as gathering real‐world evidence (e.g. through registries) required for repurposing applications to regulators.

In response, the DoH identified several actions it may take to facilitate medicine repurposing, including prioritising reviews, waiving or reducing application and evaluation fees, providing coordinated TGA and PBAC technical support and assisting with collating clinical evidence. Another proposal was to allow sponsors to apply collectively to share fees and financial risks and benefits.

However, the question of how to appropriately incentivise industry to repurpose generic medicines remains challenging and needs to balance potential public good of a new listing with the cost of subsidising a generic during a period of market exclusivity. For higher‐cost generics such as rituximab, the cost is borne by payers, but for generics that are well below PBS co‐payment thresholds, this may also result in higher out‐of‐pocket costs for patients. For example, in 2009 the U.S. Food and Drug Administration (FDA) gave URLPharma 3 years of market exclusivity for showing that a lower dose regimen of colchicine (Colcrys) was as effective as, and safer than, the recommended higher dose regimen of colchicine.^9^ The alternative would have been for prescribers simply to use a lower dose of the low‐cost generic formulation that was already available and would incur substantially lower costs to payers and patients.

### Supporting public sponsors

There are a few notable examples of older off‐patent medicines advancing through the regulatory process without commercial sponsor support but rather driven by consumer and clinician advocacy groups. An Australian example is recombinant human growth hormone, also known as somatropin, for adult growth hormone deficiency (GHD). Although the use of somatropin in adults with severe GHD was TGA registered, guideline recommended and subsidised in several other countries, it lacked public subsidy in Australia. The result was that patients were either not treated, relied on their local district medicine committee to grant compassionate access or paid costs out‐of‐pocket. This prompted the Endocrine Society of Australia and the Australasian Paediatric Endocrine Group to submit ‘public interest’ application for subsidy, for which the application fee of AU$250 000 was waived.^10^, ^11^ Although this was ultimately successful in August 2017, considerable additional resources, technical understanding and support from a generic manufacturer were still required to achieve subsidy for this indication.

International examples of successful medicine repurposing by non‐pharmaceutical company organisations include moxidectin, thalidomide and dimethyl fumarate. Moxidectin was discovered in the 1980s and used as a veterinary anti‐helminthic (worming) medicine. It was repurposed by Medicines Development for Global Health, a Melbourne‐based not‐for‐profit organisation, with support from the WHO and funding from the Global Investment Fund. They were awarded FDA priority review vouchers, which were used to expedite review, and proceeds from the sale of vouchers funded medicine development. Ultimately moxidectin was approved in 2018 to treat river blindness caused by Onchocerca,^12^ making Medicines Development for Global Health the first not‐for‐profit company in the world to register a medicine with the FDA. Thalidomide was FDA‐approved for treating erythema nodosum leprosum in 1998 based on published and non‐published studies funded by the WHO.^13^ Dimethyl fumarate, initially used as a fungicide to prevent the growth of mould on furniture, was subsequently repurposed for the treatment of multiple sclerosis and registered with the FDA in 2013 on the basis of investigator‐driven clinical trials.^7^ More recently, psychedelic‐assisted therapies for public good have drawn the support of not‐for‐profit organisations such as the Multidisciplinary Association for Psychedelic Studies (for MDMA) and Usona Institute (psilocybin), which are predominantly philanthropically funded and effectively acting as sponsors for FDA applications.

### Other potential facilitators of repurposing

Public–private partnerships, for instance between industry, academic, regulatory, clinician and patient bodies, are an appealing option to support the development and regulation of repurposed medicine candidates and sharing of the financial burdens of evidence generation. For example, National Health and Medical Research Council (NHMRC) and MRFF partnership funding could be leveraged to allow academics and industry to cover the ‘last mile’ to registration and listing together. Moreover, there is enormous untapped potential for the generation of real‐world safety and effectiveness evidence by linkage of administrative and registry databases. International examples include CNODES and Sentinel in Canada and the United States. Historically there have been many barriers to the availability of such data in Australia, but there are notable initiatives aimed at improving this access.^14^ Even when potential repurposing targets are identified, the full sponsor cost recovery model of TGA funding prohibits any additional, non‐sponsor‐funded activities such as gathering data and evaluation for registration. Hence, the TGA may benefit from public TGA funding to actively pursue repurposing.

Many older off‐patent medicines with substantial potential for therapeutic and public good are not being repurposed because of multiple barriers, leading to risks, costs and inequities in access to patients. Part of addressing these barriers involves appropriately financially incentivising Industry sponsors, but it is important to get this balance right. This means avoiding incentives to modify or reformulate existing generics with limited additional therapeutic benefits that can then be patented and marketed at even higher costs. In cases where industry cannot be appropriately incentivised, this may also involve exploring non‐traditional routes to registration and listing of existing generic medicines. This might include government supporting public sponsors and/or partnerships between generic manufacturers, government, academia, patient advocacy groups and clinicians to meet repurposing requirements.
